# Triptolide avoids cisplatin resistance and induces apoptosis via the reactive oxygen species/nuclear factor-κB pathway in SKOV3^PT^ platinum-resistant human ovarian cancer cells

**DOI:** 10.3892/ol.2013.1524

**Published:** 2013-08-12

**Authors:** YAN-YING ZHONG, HE-PING CHEN, BU-ZHEN TAN, HAI-HONG YU, XIAO-SHAN HUANG

**Affiliations:** 1 The Key Laboratory of Basic Pharmacology, School of Pharmaceutical Science, Nanchang University, Nanchang, Jiangxi 330006, P.R. China; 2 Department of Obstetrics and Gynecology, The Second Affiliated Hospital of Nanchang University, Nanchang, Jiangxi 330006, P.R. China

**Keywords:** triptolide, reactive oxygen species, nuclear factor-κB, platinum resistance, ovarian cancer

## Abstract

An acquired resistance to platinum-based drugs has emerged as a significant impediment to effective ovarian cancer therapy. The present study explored the anticancer mechanisms of triptolide (TPL) in SKOV3^PT^ platinum-resistant human ovarian cancer cells and observed that TPL activated caspase 3 and induced the dose-dependent apoptosis of the SKOV3^PT^ cells. Furthermore, TPL inhibited complex I of the mitochondrial respiratory chain (MRC) followed by an increase of reactive oxygen species (ROS), which further inhibited nuclear factor (NF)-κB activation and resulted in the downregulation of anti-apoptotic proteins, Bcl-2 and X-linked inhibitor of apoptosis protein (XIAP). Notably, the pre-treatment with N-acetyl-L-cysteine (NAC) abolished the TPL-induced ROS generation, NF-κB inhibition and cell apoptosis, but did not affect the inhibitory effect of TPL on complex I activity. These results suggested that TPL negatively regulated the NF-κB pathway through mitochondria-derived ROS accumulation, promoting the apoptosis of the SKOV3^PT^ cells. Furthermore, TPL synergistically enhanced the cytotoxicity of cisplatin against platinum-resistant ovarian cancer cells. Collectively, these findings suggest that TPL is able to overcome chemoresistance and that it may be an effective treatment for platinum-resistant ovarian cancer, either alone or as an adjuvant therapy.

## Introduction

Ovarian cancer is currently the leading cause of mortality among gynecological malignant tumors, with epithelial ovarian cancer (EOC) being the most common, accounting for >85% of all cases (1). The majority of ovarian cancers are diagnosed at an advanced stage, mostly due to a lack of effective screening strategies and difficulties in obtaining a diagnosis (2). Despite the progress that has been made in prolonging remission by the combination of surgical resection and platinum-based chemotherapy, the overall survival of patients with advanced disease is rarely >30%. The poor prognosis in the treatment of ovarian cancer is mainly attributed to chemoresistance (3). Tumor cells may dampen the cytotoxic effects of anticancer drugs via several mechanisms, including increased drug efflux, drug inactivation, alteration in the drug target and increased DNA repair (4,5). As a result, efforts have been directed towards the development of novel agents in an attempt to ameliorate the lethality of this malignancy.

Recent studies on the chemoresistance of ovarian cancer have indicated that a decreased susceptibility of the cancer to apoptosis is strongly associated with drug resistance. Constitutively activated nuclear factor (NF)-κB may be critical in the development of drug resistance in ovarian cancer cells (6). NF-κB is known to suppress apoptosis through the induction of anti-apoptotic proteins, including Bcl-2 and X-linked inhibitor of apoptosis protein (XIAP), leading to a resistance to cancer therapy and a poor prognosis (7–9). Intriguingly, numerous anticancer drugs, including the DNA-damaging agent cisplatin, are able to simultaneously stimulate NF-κB activation, as they trigger the cell death process in neoplasm cells (7,8,10). Therefore, the inhibition of NF-κB may be useful in increasing the sensitivity of cells to chemotherapy-dependent apoptosis and reversing drug resistance in ovarian cancer.

Triptolide (TPL), a purified component extracted from *Tripterygium wilfordii* Hook f (TwHf; Lei Gong Teng), has been identified as the main active element that is responsible for immunosuppressive and anti-inflammatory properties (11). A number of *in vitro* and i*n vivo* studies have revealed that TPL exhibits a wide spectrum of anticancer effects toward various cancer models (12–17). However, the underlying molecular mechanisms are complicated and remain vague. In human anaplastic thyroid carcinoma cells, TPL has been shown to induce apoptosis through the inhibition of NF-κB in a p53-independent pathway (13). TPL has also previously been shown to enhance tumor necrosis factor (TNF)-related apoptosis-inducing ligand (TRAIL)-mediated apoptosis of lung cancer cells by the inhibition of NF-κB (18). Furthermore, TPL induces the production of reactive oxygen species (ROS), leading to apoptosis in human adrenal cancer NCI-H295 cells (16). While certain NF-κB-regulated genes, including Bcl-2, play a major role in regulating the amount of ROS in the cell, ROS have various inhibitory and stimulatory roles in NF-κB signaling (19,20).

The present study aimed to investigate whether TPL sensitized platinum-resistant SKOV3^PT^ ovarian cancer cells to apoptosis, along with the molecular signaling pathway triggered by TPL in platinum-resistant cells. The study further hypothesized that TPL inactivated the NF-κB pathway through ROS accumulation, promoting the apoptosis of the SKOV3^PT^ cells.

## Materials and methods

### Materials

TPL (Sigma Aldrich Chemical Co., St. Louis, MO, USA) was dissolved as a stock solution in dimethyl sulfoxide (DMSO) and freshly diluted in 10 mM culture medium prior to use. Cisplatin, N-acetyl-L-cysteine (NAC), 3-(4,5-dimethylthiazol-2-yl)-2,5-diphenyltetrazolium bromide (MTT), Annexin V-fluorescein isothiocyanate (FITC) and propidium iodide (PI) were obtained from Sigma. 2′,7′-Dichlorodihydrofluorescein diacetate (H_2_DCF-DA) was purchased from Calbiochem (San Diego, CA, USA). The Mitochondrial Isolation kit was bought from Thermo Scientific (Pierce, Rockford, IL, USA) and the Mitochondrial Respiratory Chain (MRC) Complexes Activity Assay kits were purchased from Genmed Scientifics (Shanghai, China). Rabbit polyclonal anti-Bcl-2 (1:600), rabbit polyclonal anti-NF-κB (p65; 1:400) and goat polyclonal anti-β-actin (1:1000) antibodies were purchased from Santa Cruz Biotechnology (Santa Cruz, CA, USA) and rabbit monolonal anti-caspase 3 (1:300) and rabbit monoclonal anti-XIAP (1:500) antibodies were perchased from Cell Signaling Technology (San Diego, CA, USA).

### Cell culture

The human ovarian carcinoma-derived platinum resistant SKOV3^PT^ cell line was purchased from the American Type Culture Collection (Manassas, VA, USA). To maintain the acquired resistance to cisplatin, the cells were cultured in RPMI-1640 medium supplemented with fetal bovine serum (10%), penicillin/streptomycin (100 U/ml) and cisplatin (0.3 μg/ml) in a 5% humidified CO_2_ atmosphere at 37°C.

### Cell viability assay

Cell viability was evaluated using the MTT assay. Briefly, 1×10^4^ cells/well were seeded in 96-well microtiter plates. Following the drug treatment, the cells were incubated with 20 μl MTT (5 mg/ml) for an additional 4 h. The MTT solution in the medium was discarded and the formazan crystals, which were formed in the viable cells, were dissolved in 150 μl DMSO. The optical density of each well was measured at 490 nm using a Microplate Reader (Molecular Devices, CA, USA).

### Apoptosis analysis

Early-stage apoptosis cells that expressed phosphatidylserine on the outer layer of the cell were detected using the binding properties of fluoresceinated Annexin V (Annexin V-FITC). Briefly, the treated cells were harvested and washed twice with cold phosphate-buffered saline (PBS). The cells were suspended with a binding buffer and stained with Annexin V-FITC and PI. The cell mixture was incubated for 15 min at room temperature in the dark followed by fluorescence-activated cell sorting (FACS) cater-plus flow cytometry (Becton-Dickinson Co., Heidelberg, Germany).

### ROS detection

The changes in the intracellular ROS levels were determined using the fluorescent H_2_DCF-DA probe. Non-fluorescent H_2_DCF-DA is cell-permeable, cleaved by non-specific esterases and oxidized in the presence of ROS to form fluorescent 2′7′-dichlorofluorescein (DCF). ROS production is proportional to the fluorescence ratio of the treatment to the control. The cells were incubated with 10 μM H_2_DCF-DA for 20 min at 37°C prior to being harvested and analyzed for fluorescence intensity using flow cytometry.

### Western blotting

Following the treatment of the cells, the nuclear and cytoplasmic proteins were prepared according to the method described by Liu *et al*(21) and the protein concentrations were measured using a Bicinchoninic Acid (BCA) Protein Assay kit (Pierce). Equal amounts of proteins were electrophoresed through denaturing polyacrylamide gels, transferred onto polyvinylidene difluoride (PVDF) membranes and probed with primary antibodies against NF-κB (p65), Bcl-2, XIAP and caspase 3. Subsequent to being washed with TBST, the membranes were incubated with peroxidase-conjugated secondary antibodies for 1 h. The blots were detected with an Enhanced Chemiluminescence Detection kit (Pierce), following the manufacturer's instructions.

### Isolation of mitochondria

The mitochondria were isolated from the cultured SKOV3^PT^ cells using a Mitochondrial Isolation kit. The cells were suspended in ice-cold Mito-Cyto isolation buffer and immediately homogenized. The homogenates were centrifuged at 600 × g at 4°C for 10 min. The supernatant was transferred to a new tube and centrifuged at 11,000 × g at 4°C for 10 min. The pellet was lysed with Laemmli Buffer (Bio-Rad Laboratories, Hercules, CA, USA) to extract the mitochondrial protein. The mitochondrial protein concentration was determined by the BCA Protein Assay kit (Pierce).

### Measurement of mitochondrial complexes I, II and III activities

The activities of the MRC complexes were determined using MRC Complexes Activity Assay kits. Mitochondrial complex I (NADH-ubiquinone oxidoreductase) activity was measured by monitoring the decrease in NADH absorbance at 340 nm. The activity of complex I was calculated using the rotenone-sensitive rate and expressed as μmol/min/mg protein. Complex II (succinate-ubiquinone oxidoreductase) activity was determined in extracted mitochondria proteins through the reduction of 2,6-dichloropheno-lindophenol (DCIP) at 600 nm. The activity of complex II was calculated using the 2-thenoytrifluoroacetone-sensitive rate and the results were presented as μmol/min/mg protein. Mitochondrial complex III (ubiquinol cytochrome-c reductase) activity was measured by monitoring the reduction of cytochrome-c by ubiquinol at 550 nm and was expressed as μmol CoQH2/min/mg protein.

### Statistical analysis

Each experiment was repeated 3–4 times. The statistical analysis data were analyzed by one-way ANOVA and are presented as the mean ± SD. P<0.05 was considered to indicate a statistically significant difference.

## Results

### TPL induces apoptosis in the platinum-resistant SKOV3^PT^ ovarian cancer cell line

**Figure 1:**
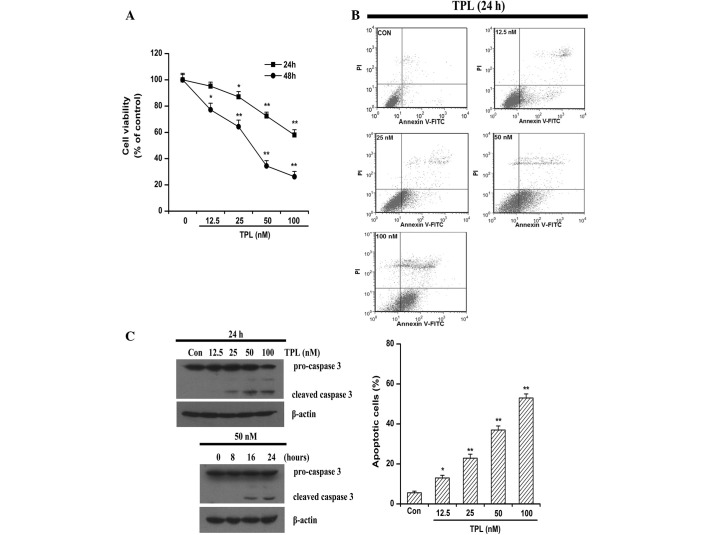
Effects of TPL on cell viability and apoptosis in SKOV3^PT^ cells. (A) SKOV3^PT^ cells were exposed to varying concentrations of TPL (0–100 nM) for 24–48 h. Cell viability was assessed by MTT assay. (B) SKOV3^PT^ cells were cultured for 24 h with varying doses of TPL (0–100 nM). Apoptosis was identified by Annexin V/PI staining and analyzed by flow cytometry. (C) Western blot analysis of caspase 3 in SKOV3^PT^ cells that were treated for 24 h with the indicated concentrations of TPL (12.5–100 nM) or at various time points with 50 nM TPL treatment. β-Actin served as a loading control. Results are presented as the mean ± SD for three independent experiments; ^*^P<0.05 and ^**^P<0.01 vs. control. TPL, triptolide; MTT, 3-(4,5-dimethylthiazol-2-yl)-2,5-diphenyltetrazolium bromide; PI, propidium iodide.

The present study evaluated the growth of the platinum-resistant SKOV3^PT^ ovarian cancer cell line under the treatment of TPL at various concentrations (0–100 nmol/l) and time points (24–48 h). The MTT assays revealed that cell viability was decreased in a dose- and time-dependent manner following exposure to TPL (Fig. 1A). The 48-h period of TPL exposure inhibited the proliferation of the SKOV3^PT^ cells with an average IC_50_ value of 34.50 nM (Fig. 1A).

To further investigate the cytotoxicity of TPL against the platinum-resistant SKOV3^PT^ cancer cells, the cells were subjected to increasing concentrations of TPL and apoptosis was assessed following 24 h by flow cytometry with Annexin V/PI staining. As shown in Fig. 1B, TPL treatment mostly induced apoptosis in the platinum-resistant cells and the proportion of AnnexinV^+^/PI^−^ (early stage of apoptosis) cells increased with the elevated TPL concentrations. Apoptosis is a tightly regulated, autonomously programmed mechanism that is finally executed by caspase 3 (22). Caspase 3 is expressed in almost all types of cells as an inactive pro-enzyme and may be activated by initiator caspase 8 or caspase 9, which subsequently cleave caspase 3 into two smaller subunits (23). Compared with the control cells, the caspase 3 activity was markedly enhanced in the cultures of the cancer cells that were treated with 25–100 nM TPL for 24 h, as evidenced by a decrease in density of the pro-form (Fig. 1C). In a time-dependent experiment, cleavage of caspase 3 was evident by 16 and 24 h of treatment (Fig. 1C). Taken together, these results indicate that the cytotoxic effect of TPL is associated with its apoptosis-inducing activity.

### ROS generation is critical for TPL-induced apoptosis

**Figure 2:**
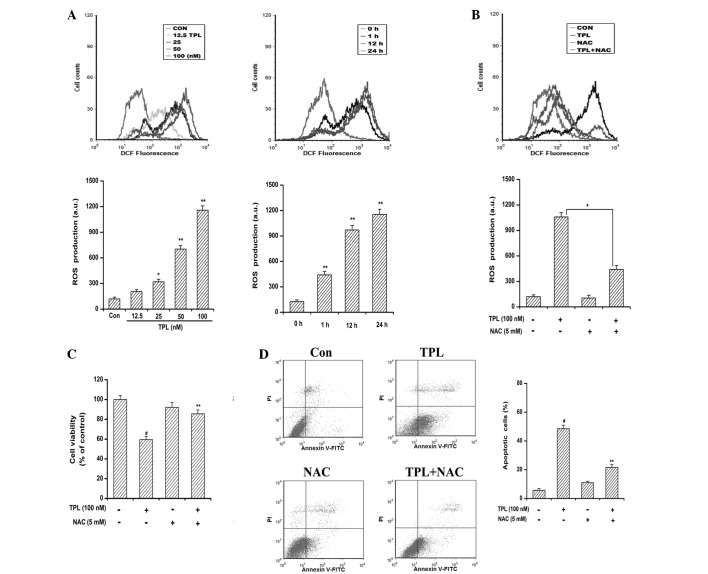
Effect of antioxidant NAC on TPL-induced apoptosis and ROS generation in SKOV3^PT^ cells. (A) Cells were incubated with TPL for the indicated concentrations (12.5–100 nM) and times (1, 12 and 24 h) and stained with H_2_DCFDA. ROS production was measured by flow cytometry. Data are presented as the mean ± SD from three independent experiments. ^*^P<0.05 and ^**^P<0.01 vs. control. (B–D) Cells were pre-incubated with 5 mM NAC for 1 h followed by incubation with 100 nM TPL for 24 h and then analyzed for (B) ROS content, (C) cell viability and (D) apoptosis. ROS content was analyzed by flow cytometry subsequent to staining the cells with H_2_DCFDA. Cell viability was determined by MTT assay. Apoptosis was measured by Annexin V/PI staining assay. Data are presented as the mean ± SD of three experiments. ^#^P<0.01 vs. control group; and ^*^P<0.05 and ^**^P<0.01 vs. TPL without NAC. NAC, N-acetyl-L-cysteine; TPL, triptolide; ROS, reactive oxygen species; H_2_DCFDA, 2′,7′-dichlorodihydrofluorescein diacetate; PI, propidium iodide.

Numerous anticancer agents exhibit antitumor activity via the ROS-dependent activation of cancer cell death (24). It has previously been reported that elevated intracellular ROS mediates TPL-induced apoptosis in human adrenal cancer NCI-H295 cells through a mitochondrial-dependent pathway (16). In the present study, to explore the involvement of ROS in TPL-induced apoptosis, the generation of ROS was measured by flow cytometric analysis using H_2_DCF-DA dye. As shown in Fig. 2A, TPL exposure resulted in a time- and concentration-dependent ROS accumulation in the SKOV3^PT^ cells compared with the DMSO-treated control cells. Significant ROS generation was observed when the cells were treated for as little as 1 h and ROS production was being maintained at a high level by 24 h, indicating a rapid and sustained generation of ROS in the TPL-treated cells. However, the production of ROS caused by TPL was greatly reduced by pre-treatment with NAC due to its ability to elevate intracellular glutathione to prevent the production of ROS (Fig. 2B). It is of note that the presence of NAC protected the cells from TPL-induced cytotoxicity (Fig. 2C). Furthermore, the flow cytometric analyses revealed that the reduction of ROS by NAC attenuated the number of TPL-induced apoptotic cells from 48.50 to 21.60% (Fig. 2D). Collectively, these data suggest that the apoptosis inducing effect of TPL is associated with ROS generation.

### Inhibition of MRC complex I is responsible for TPL-induced ROS generation

**Figure 3:**
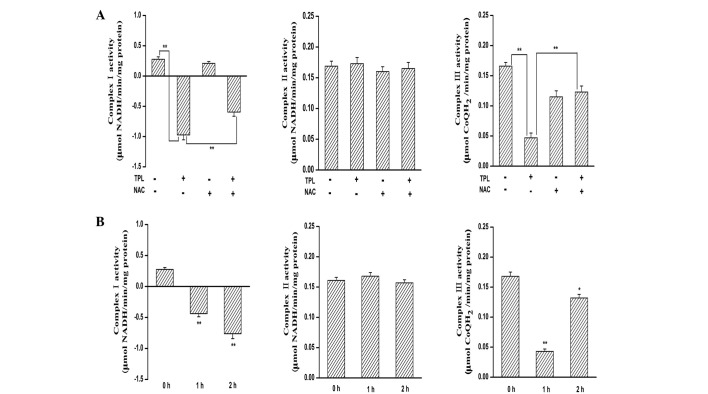
Involvement of MRC complex I in TPL-mediated ROS generation. (A) Cells were pre-treated with or without 5 mM NAC for 1 h and exposed to 100 nM TPL for 24 h. The acivities of the complexes in the mitochondria that were isolated from the SKOV3^PT^ cells were determined using MRC Complex Enzyme Activity Assay kits. Three independent experiments were performed and the data are represented as the mean ± SD (^**^P<0.01 vs. TPL without NAC). (B) Cells were treated with 100 nM TPL for the indicated time periods. Complex activities in mitochondria that were isolated from the SKOV3^PT^ cells were determined by MRC Complex Enzyme Activity Assay kits. Three independent experiments were performed and the data are presented as the mean ± SD. ^*^P<0.05 and ^**^P<0.01 vs. 0 h control. MRC, mitochondrial respiratory chain; TPL, triptolide; NAC, N-acetyl-L-cysteine.

ROS are generated during the electron transport steps of ATP production via the mitochondrial respiratory chain, involving auto-oxidation of complexes I, II and III (25). To identify the target of TPL-mediated ROS generation, the present study determined the effect of TPL treatment on the activities of MRC enzymes in the SKOV3^PT^ cells. The activities of MRC complex I and III decreased in response to the treatment with TPL (Fig. 3A). Pre-treatment with NAC substantially blocked the decreases in complex III activity by TPL (Fig. 3A). By contrast, the inhibitory effect of TPL on complex I was unaffected by NAC pre-treatment (Fig. 3A). Notably, complex II activity was not altered by TPL incubation (Fig. 3A). These results indicate that mitochondrial complex I appears to be responsible for ROS generation triggered by TPL. Additionally, the activity of MRC complex I decreased as early as 1 h following TPL exposure (Fig. 3B), which was consistent with the kinetics of ROS generation. However, the same batch and same concentration of TPL did not inhibit complex II and III activities, even following a 2-h treatment period (Fig. 3B). Accordingly, these data further lead us to speculate that TPL may cause ROS generation, at least in part, by inhibiting mitochondrial complex I activity.

### TPL treatment causes ROS-dependent suppression of NF-κB activation and cleavage of pro-caspase 3

**Figure 4:**
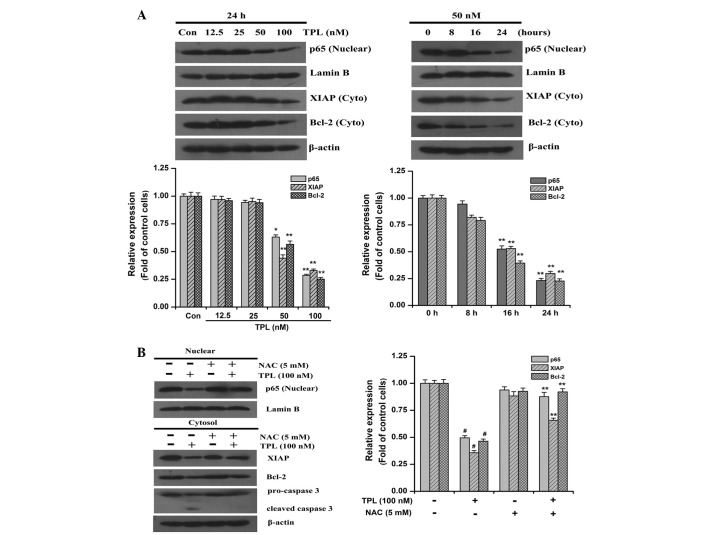
ROS-mediated TPL-induced downregulation of NF-κB/p65, Bcl-2, XIAP and cleavage of pro-caspase 3 in SKOV3^PT^ cells. (A) Western blot analysis of NF-κB/nuclear p65, XIAP and Bcl-2 following exposure to the indicated concentrations of TPL (12.5–100 nM) for 24 h or at various time points with 50 nM TPL treatment. (B) Western blot analysis of NF-κB/nuclear p65, XIAP, Bcl-2 and caspase 3 in SKOV3^PT^ cells that were pre-treated with and without 5 mM NAC for 1 h followed by 100 nM TPL for 24 h. Lamin B and β-Actin served as nuclear and cytosolic internal controls, respectively. The relative levels of protein expression are shown with the densitometric analysis and the values are expressed as the mean ± SD of three experiments. (A) ^*^P<0.05 and ^**^P<0.01 vs. the control. (B) ^#^P<0.01 vs. the control group and ^**^P<0.01 vs. TPL without NAC. ROS, reactive oxygen species; TPL, triptolide; NF-κB, nuclear factor-κB; XIAP, X-linked inhibitor of apoptosis protein; NAC, NAC, N-acetyl-L-cysteine.

NF-κB has been strongly implicated in cell proliferation, survival and chemoresistance in multiple tumors (7,8). In normal cells, NF-κB predominantly resides in the cytosol due to the inhibitory protein, IκBα, but it is translocated to the nucleus upon growth or survival stimulation (7). Therefore, the present study examined the effect of TPL treatment on the cellular localization of NF-κB (p65). As observed in Fig. 4A, the SKOV3^PT^ cells expressed substantial levels of nuclear p65 protein, implying that NF-κB is constitutively activated in these cells. Compared with the control cells, the nuclear content of p65 protein was significantly decreased in the TPL-treatment cells (Fig. 4A). The role of NF-κB in chemotherapeutic drug resistance has been associated with the induction of survival signals through the upregulation of anti-apoptotic proteins, including Bcl-2 and XIAP (8,9). As expected, the expression levels of Bcl-2 and XIAP were also decreased under TPL treatment (Fig. 4A). Notably, the TPL-induced reduction in the expression of the nuclear p65 protein and cytoplasmic Bcl-2 and XIAP proteins was inhibited by pre-treatment with NAC compared with TPL treatment alone (Fig. 4B). The effect of NAC on the activation of caspase 3 induced by TPL was subsequently examined. When the cells were treated in the presence of NAC, the cleavage of pro-caspase 3 induced by TPL was evidently suppressed (Fig. 4B). These results suggest that the inhibition of the NF-κB pathway is associated with the increased levels of intracellular ROS induced by TPL during apoptosis of platinum-resistant ovarian cancer cells.

### TPL synergistically enhances cisplatin-induced cytotoxicity in platinum-resistant cells

**Figure 5:**
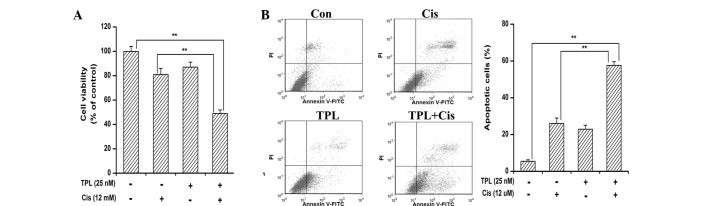
TPL sensitizes SKOV3^PT^ cells to cisplatin *in vitro*. Cells were treated with 12 mM cisplatin (Cis) or 25 nM TPL alone or in combination for 24 h. The cells were harvested to determine (A) the cell viability and (B) the percentage of apoptotic cells, as described in the Materials and methods section. The data are presented as the mean ± SD of three independent experiments (^**^P<0.01 vs. TPL+Cis). TPL, triptolide.

As the delivery of lower dose agents result in lower toxicity and an increase in patient tolerance, strategies using novel effectively safe agents or drug combinations are being increasingly investigated for overcoming chemoresistance (26,27). The present study tested whether low doses of the two drugs in combination were able to exert a synergistic anticancer effect *in vitro* compared with TPL or cisplatin alone. A dose of 12 μM cisplatin, which was the IC_50_ of cisplatin on the parental SKOV3^PT^ cells (28), had a minimal effect on cell viability in the platinum-resistant SKOV3^PT^ cells (Fig. 5A). This indicated that the SKOV3^PT^ cells were relatively resistant to cisplatin. In combination with 25 nM TPL, the inhibition rate was rapidly increased to 51.10% (Fig. 5A). TPL alone (25 nM) in the previous data revealed 12.95% cell death (Fig. 1A). The combination index (CI) of the combination was <1, suggesting that the antiproliferative effect of the combination was synergistic rather than additive. These data demonstrate that TPL is able to sensitize platinum-resistant SKOV3^PT^ cells to cisplatin. Additionally, the Annexin V apoptosis assay revealed that TPL enhanced the apoptotic effect of cisplatin from 26.07 to 57.60% (Fig. 5B). These observations demonstrate that TPL combined with cisplatin exhibits synergistic effects against platinum-resistant cells.

## Discussion

Although first-line platinum-based chemotherapy following an apparent curative resection has improved survival length, severe adverse side-effects and drug resistance have emerged as the major impediments to effective ovarian cancer therapy (29). Thus, novel strategies involving less toxic agents that are able to either enhance the antitumor effects of cisplatin or overcome chemoresistance to the drug are highly desirable.

The pleiotropic anticancer activities of TPL have attracted a great deal of research interest. TPL has been shown to possess the capacity to inhibit proliferation and induce apoptosis of various cancer cell lines *in vitro* and *in vivo*(12–17). Notably, TPL has also been identified to be effective in the induction of apoptosis in drug-resistant multiple myeloma (30) and cervical cancer (31) cells. Therefore, the present study investigated whether TPL treatment was able to exhibit a cytotoxic effect on platinum-resistant ovarian cancer cells. The results demonstrated that TPL reduced the growth of the platinum-resistant ovarian cancer cells by inducing apoptosis, evidenced by the externalization of membrane-bound phosphatidylserine and the cleavage of caspase 3. The results also showed that the addition of a low concentration of TPL greatly increased the cytotoxicity of cisplatin against the SKOV3^PT^ cells, which is consistent with previous studies (30,31).

The intracellular redox status, regulated by the production of ROS, greatly contributes to the regulation of cell survival and death (32). Oxidative stress is the condition arising from an imbalance between the production of intracellular ROS and the ability of cells to defend themselves against them (33). Although cancer cells become well adapted to persistent intrinsic oxidative stress, a further increase in ROS above the toxic threshold level may result in cell death (34). It is noteworthy that numerous commonly used chemotherapy agents, including cisplatin and etoposide, may trigger the ROS-dependent activation of apoptotic cell death (35,36). However, continuous cisplatin treatment may reduce cellular ROS levels and cancer cells containing reduced ROS may become drug resistant cells (37). Furthermore, an elevation of the cellular ROS level by exogenous ROS generation in combination with cisplatin resensitizes drug-resistant cancer cells (37). Several studies have attributed ROS generation to the pro-apoptotic effect of TPL in various cell types (16,38,39), which is in agreement with the findings of the present study.

Growing evidence supports a role for ROS in the modulation of signaling pathways, which are necessary for cell proliferation, differentiation and cancer metastasis (34,40). However, prolonged and high levels of ROS may be indicative of the stimulation of a cellular death signal via activating cell surface death receptors or acting directly on the mitochondria (41). Although a possible contribution of ROS has been observed in the apoptotic response to TPL, the mechanism by which TPL treatment causes ROS generation is unclear. Mitochondria are a major source of cellular ROS, particularly through electron leakage from the respiratory complexes (25). The inhibition of MRC complex activity is capable of leaking electrons to react with molecular oxygen, resulting in the formation of ROS (42,43). The present study demonstrated that ROS generation by TPL in platinum-resistant cancer cells occurs through MRC complex I. The activity of MRC complex I decreased following TPL treatment and NAC did not reverse the inhibition (Fig. 3A). Furthermore, the pattern of TPL-mediated ROS generation closely mirrored the inhibition of complex I activity (Fig. 3B). These results are consistent with a previous study in which celestrol induced ROS-dependent cytotoxicity by targeting MRC complex I (43). The precise mechanism of the TPL-mediated inhibition of complex I activity remains to be elucidated.

NF-κB signaling is one of the major pathways responsible for the platinum resistance of ovarian cancer, as reflected by the fact that its basal activity is significantly increased in platinum-resistant Caov-3 cells compared with A2780 platinum-sensitive cells (44). In cisplatin-resistant Caov-3 ovarian cancer cells, the inhibition of NF-κB activity by treatment with specific NF-κB nuclear translocation inhibitors (SN-50) or by the transfection of p50 ΔNLS, which lacks the nuclear localization signal domain, increased the efficacy of cisplatin-induced apoptosis (44). TPL has been identified as a novel NF-κB inhibitor and has been shown to increase the efficacy of 5-fluorouracil (FU) and TNF in cancer cells through the inhibition of NF-κB activity (38,45). The present study demonstrated that the SKOV3^PT^ cells contained substantial levels of nuclear p65 protein, which implies that substantial NF-κB activity may confer survival in these cells. The data from this study revealed that TPL blocked the transactivation of p65. Therefore the inhibition of NF-κB may account for TPL-induced cell death. Previous studies have shown that acquired cisplatin resistance in ovarian cancer is correlated with an increased expression of Bcl-2 and XIAP (46,47), which are regulated by NF-κB. The present study observed that TPL treatment led to a reduction in the expression of Bcl-2 and XIAP, which is consistent with a previous study (45). Accordingly, the inactivation of the NF-κB survival pathway may be a significant molecular mechanism contributing to the cisplatin resistance reversing effect of TPL.

NF-κB is a redox-sensitive transcription factor (48). NF-κB may be activated by ROS, resulting in the transcriptional activation of a variety of genes that are involved in cell transformation, proliferation and angiogenesis (19,49). Notably, the antioxidant, NAC, effectively blocked the intracellular ROS induced by TPL and simultaneously suppressed the reduction of p65, Bcl-2 and XIAP expression and the activation of caspase 3. These data indicate that the apoptotic effect of TPL on cancer cells through the accumulation of intracellular ROS, may function upstream of NF-κB and caspase 3. It is highly likely that the presence of oxidative stress may be decisive for the ability of TPL to inhibit NF-κB in the present study. Korn *et al*(50) reported that H_2_O_2_ was capable of inhibiting TNF-induced NF-κB activation in lung epithelial cells by the reduction in inhibitor of NF-κB kinase (IKK)-β activity through the oxidation of cysteine residues in the IKK complex. IKKβ inactivation through the oxidation of IKKβ on cysteine 179 has also been shown in arsenite treatment, leading to a reduction in NF-κB signaling (51). Further studies are required to experimentally explore these possibilities.

Altogether, the present study offers the first evidence that ROS that are produced in response to TPL treatment via a marked inhibition of mitochondrial complex I lead to NF-κB inactivation and initiate caspase 3-mediated apoptosis in platinum-resistant cancer cells. Furthermore, TPL acted co-operatively with cisplatin to induce apoptosis in the platinum-resistant cells. Further *in vivo* experiments may aid in the confirmation of the therapeutic efficacy of this agent for female patients with platinum-resistant ovarian cancer.
